# Quality of outpatient clinical notes: a stakeholder definition derived through qualitative research

**DOI:** 10.1186/1472-6963-12-407

**Published:** 2012-11-19

**Authors:** Janice L Hanson, Mark B Stephens, Louis N Pangaro, Ronald W Gimbel

**Affiliations:** 1 Departments of Medicine, Pediatrics & Family Medicine, Uniformed Services University of the Health Sciences, Bethesda, Maryland, USA; 2 Department of Pediatrics, University of Colorado School of Medicine, 13123 East 16th Ave., B-158, Aurora, Colorado, 80045, USA; 3 Department of Family Medicine, Uniformed Services University of the Health Sciences, 4301 Jones Bridge Road, Bethesda, Maryland, 20814, USA; 4 Department of Medicine, Uniformed Services University of the Health Sciences, 4301 Jones Bridge Road, Bethesda, Maryland, 20814, USA; 5 Department of Biomedical Informatics, Uniformed Services University of the Health Sciences, 4301 Jones Bridge Road, Bethesda, Maryland, 20814, USA

**Keywords:** Clinical documentation, Outpatient notes, Physician notes, Quality, Patient perspectives, Medical records, Health care records, Electronic health record

## Abstract

**Background:**

There are no empirically-grounded criteria or tools to define or benchmark the quality of outpatient clinical documentation. Outpatient clinical notes document care, communicate treatment plans and support patient safety, medical education, medico-legal investigations and reimbursement. Accurately describing and assessing quality of clinical documentation is a necessary improvement in an increasingly team-based healthcare delivery system. In this paper we describe the quality of outpatient clinical notes from the perspective of multiple stakeholders.

**Methods:**

Using purposeful sampling for maximum diversity, we conducted focus groups and individual interviews with clinicians, nursing and ancillary staff, patients, and healthcare administrators at six federal health care facilities between 2009 and 2011. All sessions were audio-recorded, transcribed and qualitatively analyzed using open, axial and selective coding.

**Results:**

The 163 participants included 61 clinicians, 52 nurse/ancillary staff, 31 patients and 19 administrative staff. Three organizing themes emerged: 1) characteristics of quality in clinical notes, 2) desired elements within the clinical notes and 3) system supports to improve the quality of clinical notes. We identified 11 codes to describe characteristics of clinical notes, 20 codes to describe desired elements in quality clinical notes and 11 codes to describe clinical system elements that support quality when writing clinical notes. While there was substantial overlap between the aspects of quality described by the four stakeholder groups, only clinicians and administrators identified ease of translation into billing codes as an important characteristic of a quality note. Only patients rated prioritization of their medical problems as an aspect of quality. Nurses included care and education delivered to the patient, information added by the patient, interdisciplinary information, and infection alerts as important content.

**Conclusions:**

Perspectives of these four stakeholder groups provide a comprehensive description of quality in outpatient clinical documentation. The resulting description of characteristics and content necessary for quality notes provides a research-based foundation for assessing the quality of clinical documentation in outpatient health care settings.

## Background

Quality of outpatient clinical encounter notes relates to patient safety [1,2], medical education [3-5], medico-legal issues [6,7], and justification of reimbursement [6-9]. The clinical note is the primary tool used to document care, communicate plans [10,11], and provide guidance for follow-up treatment and care. Gaps in the quality of clinical documentation could, therefore, adversely affect patient care and health care outcomes. Despite the importance of documentation to clinical care, there is no agreement in the literature on a comprehensive definition of the quality of documentation within a clinical note. When identifying elements associated with quality of clinical documentation, authors commonly cite attributes such as legibility, correctness, completeness, readability, appropriateness and accuracy [12-18]. Others have focused on the importance of the structure and content of the note. As early as 1969, Dr. Lawrence Weed recommended a structured outpatient clinical note and a problem-oriented medical record [19]. Weed suggested standard elements for organizing clinical documentation, including data from symptoms, physical examination and laboratory studies, a medication list, a problem list, an assessment, and a plan of care. The resulting “subjective, objective, assessment, plan” (SOAP) format has been widely used for clinical documentation for the past 40 years [20]. While it reflected and replaced the previously common but not universal format of history, physical, lab, and plan, neither provided a description of quality beyond the implicit assertion that organization is a primary requirement of quality.

Presently, many health systems and physician practices are introducing electronic health records (EHR). This may represent a unique opportunity to improve the quality of clinical documentation. One study retrospectively comparing notes of physicians using an EHR with traditional paper records demonstrates that EHRs are superior to traditional paper charts for documenting complete patient problem and medication lists, identifying relevant patient factors when making a decision, and documenting appropriate clinical decisions [17]. A separate cross-sectional review of the content and quality of a sample of records chosen from general practitioners showed that EHR documentation was more understandable (89.2% v. 69.6%), more legible (100% v. 64.3%), more likely to have at least one diagnosis recorded (48.2 % v. 33.2%), more likely to document that anticipatory advice was given (23.75 v. 10.7%), more likely to document specialty referral (77.4% v. 59.5%), and more likely to specify drug dose (86.6% v. 66.2%) compared to documentation on paper records [13]. A cross-sectional qualitative study describing perceived impact of computerized physician documentation on faculty and resident physicians showed that the EHR improved accessibility, legibility, comprehensiveness, and organization of clinical notes [3]. This study also noted, however, that the EHR also increased redundancy of information, contributed to lengthier notes, had a poor display of information, and increased “clutter” within the medical record. In summary, it is not entirely clear whether EHR use increases or decreases overall record quality. Further complicating the discussion is the fact that operational definitions of quality have yet to be consistently validated.

Stetson et al. have developed and initially validated two versions of an instrument that evaluates the quality of inpatient clinical documentation [21,22]. In their exploratory factor analysis, they identified four distinct factors relating to quality of documentation. Factor I, notes are well-formed, clear, uncluttered, organized, structured, non-redundant, and synthesized; Factor II, notes are comprehensible, legible, coherent, useful, correct, comprehensible, and consistent; Factor III, notes are up-to-date, complete, accurate, thorough, current, relevant; and Factor IV, notes are brief, concise, succinct, and focused. The items were generated by a small group of clinicians performing inpatient (hospital-based) work and did not explicitly include opinions of other stakeholders who routinely use clinical notes, such as patients, nurses, ancillary staff or administrators. In addition, it is not clear that quality in the context of documentation for inpatient care (where patients are typically sicker and have many more individuals involved in their day-to-day care processes) translates well to the outpatient environment. While analogous, documentation and its purposes in the inpatient and outpatient environments are different and one cannot assume that aspects of quality would be the same in both environments. Furthermore, there are varied and perhaps conflicting uses for clinical documents. Notes that are deemed of high quality for billing, legal, or care coordination purposes may, for example, be deemed of low quality for communication between physicians. This argues for a comprehensive description of quality in clinical notes based on perspectives from varied stakeholder groups.

Currently, there are no standardized criteria or tools to define or benchmark quality of documentation of outpatient clinical care. Similarly, there are no research-based definitions or descriptions of quality in outpatient clinical notes. No prior studies systematically incorporate into a definition of quality the perspectives of multiple individuals who write and use outpatient clinical notes. Additionally, many outpatient practices are migrating to a delivery system based on the patient-centered medical home. This team-based approach to outpatient care requires that more individuals will be accessing patient records and documenting care accordingly. With this background in mind, we sought to develop a comprehensive definition of quality in outpatient clinical notes, incorporating perspectives from a diverse group of stakeholders—the authors of notes (clinicians), direct users of notes (nursing and support staff, administrators) and patients themselves. Each of these important stakeholder groups has specific expectations for the clinical encounter note and contributes to the description of quality. The purpose of this study is to describe in detail important elements of quality in outpatient clinical notes, incorporating the perspectives of clinicians, nurses, ancillary staff, administrators, and patients.

## Methods

We used qualitative research methods to engage participants from six health care facilities and multiple stakeholder groups.

### Research setting

We conducted our study within the Military Health System (MHS). The MHS is a large capitated health system, comparable to several of the large managed care health systems in operation within the United States (e.g., Kaiser Permanente®, Partners™ Healthcare). The MHS cares for more than 9 million beneficiaries, provides approximately 34 million visits annually [23] and is representative of American society with regard to patient demographics, common diagnoses, and medical procedures [24]. The health system includes both direct care delivery and a defined benefit (i.e., TRICARE). The billing and reimbursement process associated with MHS health care delivery is consistent with civilian-managed health systems. Like civilian systems, the MHS follows Medicare regulation and reimbursement procedures (e.g., benefit eligibility, clinical documentation, clinician sign-off on notes). All outpatient clinical visits are documented in a clinical note and coded for evaluation and management codes (E&M), healthcare common procedure coding system (HCPCS) / current procedural terminology codes (CPT) to include modifiers, and International Classification of Disease codes (ICD-9-CM). Formal follow-up billing occurs when a patient has other health insurance (e.g., retirees with civilian employment-funded insurance), when the episode of care relates to an accident or occupational injury (e.g., automobile accident), or when the patient is enrolled in Medicare. Billing for Medicare patients is applied against an established Medicare-Eligible Retiree Health Care Fund which in fiscal year 2011 was approximately $11.1 billion [25]. The MHS also leverages performance-based incentives (similar to civilian pay-for-performance programs) that provide financial incentives for achieving workload output and quality of care targets. The MHS uses a common EHR and employs clinicians and support staff who are government or contract employees.

Following Institutional Review Board and secondary-level regulatory approval for conducting the study at the six facilities, focus groups and interviews were held at two tertiary medical centers, two community hospitals and two ambulatory clinics in the eastern United States. All research participants were volunteers, at least 21 years of age, and provided written informed consent prior to participating.

### Participants, inclusion criteria and sampling strategy

We used purposeful sampling to achieve maximum diversity [26]. We continued sampling until we achieved saturation in the codes and themes identified during data analysis. We recruited research participants from four groups, which represented clinicians, nursing/ancillary staff, administrators and patients. See Table 1 for a list of the occupational titles of participants. Other sampling dimensions included gender and race/ethnicity. Each of the four groups was intentionally selected to provide different viewpoints.

**Table 1:** Descriptive characteristics of research participants

**Characteristics**	**Clinician group**	**Nurse/Ancillary group**	**Patient group**	**Administrator group**
**Number = 163**	61	52	31	19
**Disciplines/fields represented**	Physicians	Cert. nurse specialists	Patients	Quality assurance specialists
	Nurse practitioners	Nurses	Caregivers	Special needs coordinators
	Physician	Medical technologists		Patient advocates
	Assistants	Physical therapists		Patient safety managers
	Dentists	Social workers		Medical coders
	Psychologists	Pharmacists		Group practice managers
	Optometrists	Medical assistants		Medical records technicians
		Case managers		Patient administrators
		Dental assistants		
		Hospital Corpsman		
**Gender**				
Female	29 (47.5%)	39 (75.0%)	20 (64.5%)	13 (68.4%)
Male	30 (49.2%)	11 (21.2%)	11 (35.5%)	6 (31.6%)
Not reported	2 (3.5%)	2 (3.8%)	0	0
**Age (in years)**				
Mean	41.6	42.1	51	41.8
Maximum	72	62	83	61
Minimum	26	21	25	26
# participants not reporting	1	3	9	0
**Race/ethnicity**				
African American	2 (3.3%)	18 (34.6%)	8 (25.8%)	10 (52.6%)
Asian Pacific	7 (11.5%)	3 (5.8%)	0	0
Hispanic	3 (5.0%)	2 (3.8%)	3 (9.7%)	4 (21.1%)
Caucasian	48 (78.7%)	28 (53.8%)	20 (64.5%)	5 (26.3%)
# participants not reporting	1 (1.6%)	1 (1.9%)	0	0
**Data collected between**	May 2009 –December 2009	June 2009 –December 2009	July 2009 –October 2011	May 2009 –December 2009

#### Clinician group

Participants in the clinician group were licensed physicians or physicians-in-training (intern, resident, fellow), nurse practitioners, physician assistants, dentists, optometrists and psychologists. Each of these individuals was responsible as an individual author of outpatient clinical encounter notes. All were actively credentialed in clinical practice and using AHLTA (Armed Forces Health Longitudinal Technology Application; the MHS EHR) to document clinical care. Most clinicians were from primary care environments, in particular, internal medicine and family medicine.

#### Nursing/ ancillary care group

Participants in this group were nurses, medical technologists, physical therapists, social workers, pharmacists, case managers, medical assistants, dental assistants, or military medical technicians. These individuals commonly read clinical encounter notes as part of their professional duties, using the information in the notes to provide or coordinate patient care. All had completed EHR training and were familiar with the use of AHLTA.

#### Administrators

Participants in this group were actively employed in an administrative capacity at one of the study sites. Administrators routinely reviewed patient clinical encounter notes as part of their official administrative duties. Related occupational positions included billing staff and supervisors, medical records technicians and their supervisors, quality improvement staff, patient safety managers, group practice managers, and patient administration staff members.

#### Patients/caregivers

Participants in the patient and patient caregiver group were actively receiving care at one of the study sites. All had attended at least one outpatient clinical visit during the past 12 months as either a patient or an individual responsible for assisting with patient care in the home.

### Qualitative research methods

We used the grounded theory approach to advise our study design, data collection and analysis. This approach derives theory from data which have been systematically gathered and repeatedly analyzed [27,28].

Data were collected through focus groups and interviews at the six clinical sites. Most focus groups were small (7 to 11 participants) and facilitated by two investigators who de-briefed after each focus group to discuss emerging themes, focus group process and needed adaptations in the focus group guide. Focus groups and interviews followed the semi-structured guide in Appendix 1 and a modified interview guide for patients and caregivers (Appendix 2). All sessions were audiotaped with two recorders and transcribed by contracted transcription professionals. Transcriptions were created in .txt files using Microsoft® Word and linked to HyperResearch^TM^[29] for organization of data during qualitative analysis.

### Qualitative data analysis

We applied open, axial and selective coding techniques to the focus groups and interview transcripts for analysis [28]. Two investigators (JH and RG) developed an initial set of codes (open coding), applied them to 6 transcripts, identified and resolved all coding disagreements, and revised the codes. A third investigator (MS) then reviewed the coding of all 6 transcripts and recommended revisions to the names and definitions of the codes. After grouping the codes in three organizing themes (axial coding), all four investigators discussed and refined the coding scheme to explain the entire dataset. All transcripts were coded, with at least 2 investigators reviewing each transcript; all disagreements were discussed and resolved. During all coding and reviewing of coding, all investigators added memos to the transcripts. The theme describing characteristics of quality in a note was identified as the main organizing theme for the dataset (selective coding). We ensured that the data reached saturation (i.e., no new themes were emerging) for each of the four groups of participants (clinicians, nurses/ancillary staff, administrators, and patients) before we stopped collecting data. The transcripts were coded as one complete dataset with one integrated set of codes and themes. After we completed coding, we examined which stakeholder groups’ transcripts contained which codes and themes, in the data as organized in the HyperResearch™ software.

## Results

A total of 163 research participants representing the four targeted groups (clinicians, nurse/ancillary, patients, and administrators) participated in focus group sessions or interviews between May 2009 and October 2011 (Table 1). These included 61 clinicians in 9 focus groups, 52 nurses and ancillary care providers in 6 focus groups, 19 administrators in 5 focus groups and 31 patients and caregivers in 1 focus group plus interviews with individuals or couples. The distribution across groups with respect to gender was relatively equal, with the exception of the nurse/ancillary and administrator groups, who had more females (75% and 68.4% respectively). The age distribution across groups was balanced (mean age between 41.6 years and 41.8 years), with exception of the patient group, which was slightly older (mean age 51 years, although young adult patients were represented in this group). Participants self-identified as African-American, Asian or Pacific Islander, Hispanic and Caucasian.

We identified three major organizing themes relating to quality of outpatient clinical documentation; each theme includes specified sub-themes (Table 2). The first organizing theme identifies *characteristics* associated with quality within a clinical note. With the exception of three characteristics (ease of translation into codes, prioritized and relevance), all four stakeholder groups discussed each of the characteristics in this theme (Table 3). According to participants, a clinical note of high quality is concise, has sufficient information, and explains the clinician’s thought process and the plan of care. A quality note is also clear and understandable to patients, subsequent providers and others who might read the note. The note should contain information that is relevant, current, accurate and prioritized for action. A note of high quality is readable with appropriate font, legible handwriting, correct spelling, few or no abbreviations, and understandable syntax. The note should be organized and tell a continuous story about the patient.

**Table 2:** Themes and codes

**Characteristics of quality in a clinical note [Main organizing theme]**	
a	Conciseness *(focused; brief; not redundant)*
b	Sufficiency of information *(enough information for diagnosis, treatment, coding; pertinent details present; complete for its purpose)*
c	Explanatory *(explains clinician thought process; gives reasons for diagnosis and plan)*
d	Clarity *(clear; understandable to patients, to subsequent providers, and to other users)*
e	Relevance *(only relevant information; no extraneous information)*
f	Prioritized
g	Readability *(readable font; correct spelling; no abbreviations or only unambiguous abbreviations; readable output from EHR; legible handwriting; understandable syntax)*
h	Organization *(well-organized; logically grouped; chronological; important parts highlighted; can find the information you need easily)*
i	Continuity of story *(tells a story; written in free text with a flow that makes sense; shows continuity from referral to note and from one provider to another; internally and externally consistent; facilitates follow-up with the information provided; synthesizes information; coordinates information from different sources)*
j	Current and accurate *(has current information; up-to-date; correct; from a patient’s perspective, accuracy includes honesty and whether the note includes what the patient said)*
k	Ease of translation into codes (*diagnostic; procedural; other)*
**Content elements of the note**	
a	Patient’s complaints
b	History of the present illness *(“HPI”; “subjective”)*
c	Problem list
d	Past medical history
e	Medications list
f	Adverse drug reactions and allergies *(distinguished from side effects of medications, which is included in prognosis and expectations)*
g	Social and family history *(includes the patient’s reaction to the diagnosis or health condition)*
h	Review of systems
i	Physical findings *(pertinent positives and negatives; “objective;” vital signs)*
j	Assessment *(diagnosis; differential)*
k	Plan of care *(with goals and objectives)*
l	Follow-up information *(instructions for the patient; consults; orders; prescriptions; language and other learning barriers for patients)*
m	Author information *(name; title; discipline; date of the encounter)*
n	Patient identifiers
o	Prognosis and expectations *(includes side effects of medications)*
p	Care and education delivered
q	Information added by the patient
r	Interdisciplinary information
s	Infection alerts
t	Patient priorities
**System supports for quality documentation**	
a	Reliability and accessibility *(works when you need it; you can get into it; notes available when you need them)*
b	Interoperability *(integrated inpatient records, outpatient records, emergency department and pharmacy; information linked between facilities)*
c	Structures input well *(ease of writing; links to templates; time efficient; limits copying and pasting; easy to correct errors)*
d	Structures output well *(for ease of viewing and reading; useable display; links to patient’s history—medical, surgical, medications, allergies, problem list; links information between different notes; you can find needed information about a patient; links from diagnosis to occupational exposure; works well for security and patient privacy)*
e	Time *(time with patient; time to write notes)*
f	Ancillary staff *(available to help in clinic)*
g	Relationship with patient *(good relationship facilitates good note)*
h	Workstations *(place to see patients and write notes is convenient)*
i	Can correct errors
g	Patient computer *(for patient to answer questions)*
k	Education and training *(sufficient training on how to write notes in the EHR and use templates or formats)*

**Table 3:** Sources of data contributing to “characteristics of quality in a clinical note”

**Characteristics**	**Clinicians**	**Nurse/ancillary**	**Patients**	**Administrators**
**Conciseness***(focused; brief; not redundant)*		✓	✓	✓
**Sufficiency of information***(enough information for diagnosis, treatment, coding; pertinent details present;, complete for its purpose)*	✓	✓	✓	✓
**Explanatory***(explains clinician thought process; gives reasons for diagnosis, plan)*	✓	✓	✓	✓
**Clarity***(clear or unclear; understandable to patients, to subsequent providers, to other users)*	✓	✓	✓	✓
**Relevance***(only relevant information; no extraneous information)*	✓	✓		
**Prioritized**			✓	
**Readability***(readable font; correct spelling; no abbreviations or only unambiguous abbreviations; readable output from EHR; legible handwriting; understandable syntax)*	✓	✓	✓	✓
**Organization***(well-organized; logically grouped; chronological; important parts highlighted; can find the information you need easily)*	✓	✓	✓	✓
**Continuity of story***(tells a story; written in free text with a flow that makes sense; shows continuity from referral to note and from one provider to another; internally and externally consistent; facilitates follow-up with the information provided; synthesis)*	✓	✓	✓	✓
**Current and accurate***(has current information; up-to-date; correct; from a patient’s perspective, accuracy includes honesty and whether the note includes what the patient said)*	✓	✓	✓	✓
**Ease of translation into codes***(diagnostic; procedural; other)*	✓			✓

The concept of “continuity of story” was noted repeatedly in all four stakeholder groups. Comments coded “continuity of story” encompassed telling a coherent story about the patient from one visit to the next, as well as facilitating the linkage of one encounter note to related information and encounters. A continuous story facilitates follow-up, synthesizes patient information and coordinates information from different sources. Stakeholder emphasis in the *characteristics* theme varied. For example, clinicians, nurses and administrators discussed “current and accurate” primarily in relation to whether the information in the note was updated for the patient’s current situation and was factually correct. Patients, in contrast, discussed “accuracy” in terms of whether the clinician included “honest” information and incorporated the information that the patient told the clinician. The final code in the organizing theme of *characteristics*, “ease of translation into codes,” was emphasized by administrators and mentioned by clinicians, but not mentioned by nurses or patients.

The second organizing theme is *content*, which details the elements that the four stakeholder groups expect in a quality clinical note. Within this theme, there was more variation in the codes among the four groups. Different groups emphasized aspects of the note that particularly facilitated their individual roles (Table 4). Although terminology sometimes differed, all four groups agreed that a quality note included the following elements: patient complaints; history of the patient’s current illness; a list of the patient’s problems; the past medical history; a medication list; social and family history (patients emphasized this more than the other groups and included their emotional reactions to diagnoses and health conditions); assessment; plan of care; information needed to understand and implement follow-up care. Clinicians and nurses included adverse drug reactions and allergies; clinicians and administrators included a review of systems; clinicians, nurses and administrators included author information and patient identifiers. Nurses stressed that a quality note should include documentation of care and education delivered to the patient, information that patients add to their own notes, interdisciplinary information from the various health care providers involved in the patient’s care, and infection alerts when a patient’s care required attention to infection control. Patients emphasized the importance of including the patient’s priorities and a description of the patient’s prognosis regarding what they could expect in the future, including the effects and side-effects of treatment.

**Table 4:** Sources of data contributing to “content elements of the note”

**Content elements of the note**	**Clinicians**	**Nurse/ancillary**	**Patients**	**Admin.**
**Patient’s complaints**	✓	✓	✓	✓
**History of present illness***(“HPI;” “subjective”)*	✓	✓	✓	✓
**Problem list**	✓	✓	✓	✓
**Past medical history**	✓	✓	✓	✓
**Medications list**	✓	✓	✓	✓
**Adverse drug reactions and allergies***(distinguished from side effects of medications, which is included in prognosis and expectations)*	✓	✓	✓	✓
**Social and family history***(includes the patient’s reaction to the diagnosis or health condition)*				
**Review of systems**	✓	✓	✓	✓
**Physical findings***(pertinent positives and negatives; “objective;” vital signs)*	✓	✓	✓	✓
**Assessment***(diagnosis; differential)*	✓	✓	✓	✓
**Plan of care***(with goals and objectives)*	✓	✓	✓	✓
**Follow-up information***(instructions for the patient; consults; orders; prescriptions; language and other learning barriers for patients)*	✓	✓	✓	✓
**Author information***(name; title; discipline; date of encounter)*	✓	✓	✓	✓
**Patient identifiers**	✓	✓	✓	✓
**Prognosis and expectations***(includes side effects of medications)*				
**Care and education delivered**		✓		
**Information added by the patient**		✓		
**Interdisciplinary information**		✓		
**Infection alerts**		✓		
**Patient priorities**			✓	

The third organizing theme discusses *healthcare system contributions* to the quality of clinical notes. Collectively, the four groups addressed the medical records system, education and training, and the clinical care environment. Clinicians and nurses discussed systems issues in the most detail. According to the stakeholders, the records system supported quality notes through reliability and accessibility, interoperability (between different aspects of the care system), data input structures, opportunity to correct errors and data output structures. Other system issues relating to quality documentation included adequate time to spend with patients and write notes, adequate ancillary support, and efficient workstations for clinicians, staff and/or patients to contribute to their notes. Patients and administrators commented on the appearance of copied notes, patients noted how time and relationship with clinicians affected the note, and administrators noted the importance of education for clinicians, particularly in terms of learning how to efficiently use EHR systems and include information needed for coding (Table 5).

**Table 5:** **Sources of data contributing to** “**system supports for quality documentation”**

**System supports**	**Clinicians**	**Nurse/ancillary**	**Patients**	**Admin.**
**Reliability and accessibility***(works well when you need it; you can get into it; notes available when you need them)*	✓	✓		
**Interoperability***(integrated inpatient records, outpatient records, emergency department and pharmacy; information linked between facilities)*	✓	✓		
**Structures input well***(ease of writing; links to templates; time efficient; limits copying and pasting; easy to correct errors)*	✓	✓		
**Structures output well***(ease of viewing and reading; usable display; links to patient history including medical, surgical, medication, allergy and problem list information; links information between notes; links from diagnosis to occupational exposure; works well for security and patient privacy)*	✓	✓	✓	✓
**Time***(time with patient; time to write notes)*	✓		✓	
**Ancillary staff***(available to help in clinic )*	✓			
**Relationship with patient***(good relationship facilitates good note)*	✓		✓	
**Workstations***(place to see patients and write notes that is convenient)*	✓			
**Can correct errors**		✓		
**Patient computer***(for patient to answer questions)*		✓		
**Education and training***(sufficient training on how to write notes in the EHR and use templates or formats)*	✓	✓		✓

## Discussion

We asked clinicians, nurses/ancillary staff, administrators and patients/caregivers to describe quality in clinical documentation. Three organizing themes emerged: 1) characteristics of quality in clinical note—11 key characteristics were described; 2) content elements of clinical notes—20 key elements were described; 3) system support factors for writing quality clinical notes—11 key factors were described.

The organizing theme regarding the characteristics of a note provides a collective description of quality on which the four stakeholder groups substantially agreed. The theme delineating desirable content elements within a note provides a list that could be used to measure a note’s comprehensiveness. While the four groups agreed on many important content elements (patient complaints, symptoms, problems, history, medications, assessment and plan of care), they included or emphasized some elements very differently. Nurses and patients emphasized the importance of detailed, clear, practical information about needed follow-up care, as well as information provided by the patient, such as patient entries in the note or other documentation about patient priorities and patients’ explanations of their problems. Nurses also emphasized the importance of interdisciplinary contributions to a note, to assist with continuity of story. Administrators emphasized the importance of easy translation of information in the notes to codes for medical billing purposes. The third organizing theme we identified relates to system factors that facilitate or inhibit quality clinical documentation. Issues such as data entry, clinical workflow, system interoperability, access, reliability and data output all impact the end-product in terms of quality documentation.

The inclusion of patients in our study calls to mind the work of Delbanco and colleagues in the OpenNotes project [30,31]. Most of the patients and caregivers in our study had not seen outpatient notes about their care, which is the reason we created a modified interview guide to elicit their perspectives. They were, nevertheless, able to explain what information they wanted to have in a note about them, such as their priorities for their care, and how a note could help them coordinate their own care or explain their healthcare needs to other family members. Perhaps as patients have opportunities to read their notes, as in the OpenNotes project, more conversations will emerge about what quality means to those about whom the notes are written.

The grounded theory that emerges is that our stakeholder groups agree on most characteristics of quality in a note. A high-quality outpatient clinical note is concise, explanatory, clear, relevant, prioritized, readable, organized, current and accurate. A quality note contains sufficient information for the reader to understand its rationale and tells a coherent and continuous story. Since individuals define quality in terms of content, as it relates to their role when using the note, it follows that quality content as defined by clinicians differs from that as defined by nurses, administrators and patients. Therefore, we plan to devise a quality rating instrument that encompasses multiple perspectives.

The research participants from the clinician and nurse stakeholder groups also noted that the health care records system impacts quality documentation according to reliability, accessibility, interoperability and the structure of data input and output, and that the clinical system also makes the production of quality notes more or less likely. System issues related to time, staffing, the support of clinician/patient relationships, convenient workstations, patient data entry, and education/training may also influence quality in clinical documentation. When we began this study, we tried to focus the discussion on quality of notes apart from reference to whether the record system was paper-based or electronic. The clinical participants, however, spoke from the context of their work and commented extensively about the influence of the electronic record system on the quality of notes they wrote and read. In accordance with qualitative research methods, we added 2 questions about this to the focus group guide as the study progressed (Appendix 1) and we reported themes about influence of the EHR on quality as they emerged from the analysis. Nevertheless, there are definite limitations to the insight that this study can add to the complex question of how factors related to EHR use may affect the quality of clinical documentation. This question warrants study in future research.

Our results add additional characteristics and elements of quality in clinical documentation to the existing literature. For example, the traditional structural elements in a SOAP note appear on our list of content elements as well. The clinicians in our study likely acquired this standard terminology during their medical education. Our characteristics also confirm those noted by Stetson et al. that describe quality documentation in the inpatient setting. The themes in the current study, however, describe additional aspects of quality documentation that arise in the outpatient setting, such as integration of insights and care across disciplines and incorporation of patients’ priorities in ongoing treatment plans and care. These may, of course, also be important in the inpatient setting; and they might also be identified by our study design when applied to clinical documentation for inpatients. A key additional finding from our stakeholders is that quality notes are also “explanatory” and provide “continuity of story”. “Continuity of story” relates to “narrative expressivity” as identified by Rosenbloom and colleagues in their work with clinicians [6,32]. In our study the concept arose across stakeholder groups and included connecting different aspects of the patient’s story and care, which seems to go beyond the “narrative expressivity” and “flexibility” described in Rosenbloom’s work. This continuity of story highlights that a single patient encounter occurs in a clinical context, often as one of a series of encounters. Often different disciplines and multiple healthcare professionals are involved with varying degrees as part of a patients’ longitudinal story. A single clinical note represents one clinical encounter, a ‘chapter’ in an evolving health care story in the life of the patient. The best notes, according to our groups, make clear the connections between different chapters and thereby enrich the patient’s story.

Our results also provide insight about the information that various stakeholders seek when defining quality in clinical documentation. Administrators, patients and nurses seek different information to fulfill their roles or implement the care recommendations they receive. The clinical note, therefore, contains interdisciplinary practical context that cannot be ignored in a comprehensive definition or evaluation of quality. It may be that the published literature to date has emphasized clinicians’ needs, with less focus on how the clinical note affects other healthcare providers and patients.

Our data were collected within a single healthcare system, which may be a limitation of the study. While the system is large and the sample diverse, and we believe that the literature shows the system to be comparable to other large managed care organizations [23,24], future research should replicate our study in other systems. Another limitation applies particularly to the insights put forth about how systems issues may affect the quality of clinical documentation. Since the focus of the study is quality of notes, not the systems within which notes are written, the scope of our findings for this theme is likely limited. A more thorough treatment of the impact of systems on note quality would require a focused study on this question in settings that use a variety of documentation systems.

Our research-based definition of quality in clinical documentation describes quality in a clinical note from the perspectives of those who most commonly use the note for clinical, nursing or administrative purposes and the patients who are the subject of the note. The inclusion of the voice of those who use these notes, and the patients about whom these notes are written, represents a novel contribution to the understanding of “quality” in this context. We suggest that comprehensive study of quality of clinical documentation should incorporate the perspectives of these various stakeholder groups, and that achieving quality outpatient clinical documentation requires addressing the needs described by those who use clinical notes to plan, implement and pursue care, as well as by those who write the notes and use them to document what happens in the clinical encounter.

## Conclusions

Perspectives of four stakeholder groups were necessary to provide a comprehensive description of quality in outpatient clinical documentation. The resulting description of characteristics and content necessary for quality documentation provides a research-based foundation for assessing and improving clinical documentation in outpatient health care settings.

## Appendix 1 Focus Group and Interview Topic Guide

### Study on Quality in Notes

Materials available for review

Preliminary themes from other focus groups and interviews in this study, if available

Draft list of dimensions of quality of a medical note, after preliminary analysis of initial focus groups and interviews

**Topics for discussion** (identified from the literature and discussion among investigators)

Comprehensiveness of notes; essential items to include

Accuracy/concordance with care/relevance (diagnosis, treatment, follow-up)

Intelligibility/comprehensibility/understandability/clarity

Effect on patient care

Effect on patient/clinician encounter

Quality of narratives

Speed and ease of use/timeliness

Support for medical decision-making

Organization

General, open-ended questions

(Tailor wording of the questions for each focus group or interview participant.)

What makes a quality note? OR What constitutes “goodness” or “badness” in a clinical note?

What diminishes the quality of a note?

What do you consider essential content for a clinical note?

How does format (written system, electronic system, templates, pick-lists) help improve the quality of notes?

How does format (written system, electronic system, templates, pick-lists) hurt the quality of notes?

What training about writing notes might improve quality?

What tips have you discovered that help make the quality of notes better?

Additional questions…

…will be developed based on experience in early focus groups and interviews of the study.

…will be asked during focus groups to clarify and explore comments offered by study participants. These are the “probing questions” of focus group methodology.

## Appendix 2 Patient/Family Member Interview Topic Guide

### Study on Quality in Notes

Materials available for review

Preliminary themes from other focus groups and interviews in this study

Draft list of dimensions of quality of a medical note, after preliminary analysis of initial focus groups and interviews

**Topics for discussion** (identified from the literature and discussion among investigators)

Comprehensiveness of notes; essential items to include

Accuracy/concordance with care/relevance (diagnosis, treatment, follow-up)

Intelligibility/comprehensibility/understandability/clarity

Effect on patient care

Effect on patient/clinician encounter

Quality of narratives

Speed and ease of use/timeliness

Support for medical decision-making

Organization

**Note**: Additional questions, “probing questions,” may be asked to clarify what the interview participant means and to explore new topics that the participant raises during the interview.

**Open-ended interview questions**:

1. Have you seen notes that your doctor or another health care provider has written about you?

2. If yes:

a. What do you find helpful about these notes?

b. What makes these notes good?

c. What makes these notes helpful for you?

d. What makes these notes useful for other health care providers?

e. What makes these notes less helpful or less useful to you or to anyone else?

3. If no:

a. What do you think your doctors and your other health care providers write about you and your health?

b. What do you think would make these notes helpful or useful?

c. What do you think would make these notes unhelpful or not as useful as they could be?

4. Can you think of anything that would make the notes that your health care providers write about you more useful to you?

Comments on tentative themes from other groups:

Have a copy of the tentative themes for interviews and focus groups, folded in half.

1. Show the participant the “content” themes and say, “The health care providers that we have talked to have told us that they look for these things in a note about a patient.”

a. What do you think about this list of things that might be written in your medical record?

b. Which of these things do you think might be most important to have in a health care provider’s note about you?

c. Is there anything on this list that you wish would NOT be written about you?

d. Is there anything else that you want your health care providers to write about?

2. Show the participant the “characteristics” themes and say, “The health care providers that we have talked to have told us that they want a note about a patient to have these characteristics.”

a. Which of these things do you think would be most important for a note that a health care provider writes about you?

b. Are there any other characteristics that you can think of that would make the notes that your health care providers write about you excellent notes?

## Abbreviations

EHR: Electronic health record; MHS: Military health system; AHLTA: Armed forces health longitudinal technology application.

## Competing interests

The views expressed in this paper reflect those of the authors and not necessarily those of the Uniformed Services University of the Health Sciences, the United States Department of Defense or the United States federal government. The authors declare that they have no competing interests.

## Authors’ contributions

Study concept and design: JLH, MBS, LNP, RWG. Data collection: JLH, MBS, LNP, RWG. Qualitative analysis and interpretation of data: JLH, MBS, LNP, RWG. First draft of manuscript: RWG, JLH. Critical revision of the manuscript for important intellectual content and interpretation: JLH, MBS, LNP. Administrative, technical and material support: RWG. Review and approval of the manuscript: JLH, MBS, LNP, RWG. All authors read and approved the final manuscript.

## Authors’ information

Dr. Hanson brings expertise in qualitative research design and methods, medical education and communication. Dr. Stephens is an experienced family physician and medical educator with additional expertise in use of electronic medical records. Dr. Pangaro is an experienced internist, geriatrician and endocrinologist with additional expertise in medical education and evaluation. Dr. Gimbel brings expertise in quality improvement and biomedical informatics.

## Pre-publication history

The pre-publication history for this paper can be accessed here:

http://www.biomedcentral.com/1472-6963/12/407/prepub
